# Transcriptomic study in explanted liver from a patient with acute intermittent porphyria

**DOI:** 10.1002/jmd2.12329

**Published:** 2022-09-11

**Authors:** Jordi To‐Figueras, Esther Titos, Paula Aguilera, Alba Díaz, Javier Muñoz‐Luque, Irene Madrigal, Celia Badenas, Mercè Torra, Constantino Fondevila, Jordi Colmenero

**Affiliations:** ^1^ Biochemistry and Molecular Genetics Unit, Hospital Clinic, Institut de Recerca Biomedica August Pi Sunyer (IDIBAPS) University of Barcelona Barcelona Spain; ^2^ Dermatology Unit, Hospital Clinic, IDIBAPS University of Barcelona Barcelona Spain; ^3^ Pathology Unit, Hospital Clinic, IDIBAPS University of Barcelona Barcelona Spain; ^4^ CIBERehd University of Barcelona Barcelona Spain; ^5^ CIBER of Rare Diseases (CIBERER), Instituto de Salud Carlos III Madrid Spain; ^6^ Hospital Universitario La Paz, IdiPAZ, CIBEREHD Madrid Spain; ^7^ Liver Transplant Unit, Liver Unit, Hospital Clínic, IDIBAPS University of Barcelona Barcelona Spain

**Keywords:** acute porphyria, heme‐synthesis pathway, liver transplant, RNA, transcriptomics

## Abstract

Acute intermittent porphyria (AIP) is a rare disease caused by a deficiency of hydroxymethylbilane synthase (HMBS), the third enzyme of the heme‐synthesis pathway. Decreased enzymatic activity in the liver induces an overproduction of heme‐precursors and acute neurological attacks. We report a 36‐years‐old female with AIP with a long‐term history of severe, disabling, recurrent attacks, who underwent curative liver transplantation. Tissue samples from the explant were obtained for transcriptome analysis. Whole RNA was extracted and 16 gene‐transcripts were selected and investigated by quantitative polymerase chain reaction. These included nine genes encoding enzymes that consecutively catalyze heme‐synthesis and catabolism in the liver (*ALAS1*; *ALAD*; *HMBS*; *UROS*; *UROD*; *CPOX*; *PPOX*; *FECH*; *HMOX1*). Additionally, we studied genes related to inflammation (*IL6*; *TNF*) insulin signaling (*PGC‐1α*; *IGF‐1*; *FOXO‐1*) and tryptophan metabolism (*TDO2*; *IDO*). Transcripts of eight house‐keeping genes were co‐measured for normalization. All transcripts were also measured in five control samples from healthy living liver donors. The transcriptome of the controls showed important differences between the various genes, with the first two genes of the heme‐synthesis pathway, *ALAS1* and *ALAD* showing strikingly high mRNA levels compared to the consecutive HMBS gene. Transcripts of several genes significantly differed in the AIP liver compared to controls. Transcripts of *HMOX1* and *UROS* were increased in the AIP liver whereas transcripts of *UROD*; *CPOX*, *PPOX*, and *TDO2* were decreased. *ALAS1* expression was not increased, possibly due to hemin administered to the patient before transplantation. These results highlight several transcriptomic changes related to heme homeostasis in AIP.

## 1 INTRODUCTION

Acute intermittent porphyria (AIP) is a rare disease caused by a deficiency of hydroxymethylbilane synthase (HMBS), the third enzyme of the heme‐synthesis pathway.^1^ Carriers of pathogenic variants within the *HMBS* gene are at risk of presenting acute neurological attacks after external or internal stimuli induce the accumulation of 5‐aminolevulinic acid (ALA) and porphobilinogen (PBG).^2^ Several treatment options are currently available, among which intravenous human hemin (heme‐arginate) infusions and, more recently, the administration of a small interfering RNA, givosiran.^3^ However, in some patients the presentation of severe AIP may require the need for liver transplantation (LT), which has been shown to be curative, thereby confirming the hepatic origin of the disorder.^3^ The first AIP patient who benefitted from LT was reported in the UK in 2002^4^ and between 2002 and 2019, 38 AIP patients received LT in 12 European countries as comprehensively reviewed by Lissing et al.^5^

Several studies have analyzed the characteristics of the explants, focusing especially on the histopathology and iron accumulation after long‐term treatment with heme‐arginate.^5^ However, only a few of these studies investigated mRNA in the explanted tissue. Yasuda et al.^6^ published a comprehensive study of an explanted AIP liver, which included quantitation of mRNA levels of two key genes: *ALAS1* and *HMOX1*. Another study in France^7^ also studied these two genes in liver explants. However, no study has addressed the transcriptomics of the eight genes regulating the heme‐synthesis pathway in the liver of a patient with AIP.

We extracted RNA from a liver sample obtained from an AIP patient (AIP‐L) who underwent curative LT in Barcelona and analyzed the mRNA of 16 genes by quantitative polymerase reaction (qPCR; Table 1, including gene abbreviations). The set of mRNAS analyzed included transcripts of all the genes of the heme‐synthesis pathway plus additional genes indirectly associated with AIP (Table 1). The mRNA levels in the AIP‐L tissue were compared with those of five tissue samples obtained from healthy living liver donors. Here, we report on the transcriptomics of a set of genes regulating heme synthesis, degradation, and utilization all closely related to acute porphyria.

**TABLE 1:** Genes selected for the transcriptomic study with abbreviations, chromosome location, and the main physiological function/relation with porphyria

Selected genes	Symbol	Chromosome	Main function/relation to porphyria
Delta‐aminolevulinate synthase 1	*ALAS1*	3p21.2	Heme biosynthesis, first step in the liver
Aminolevulinate dehydratase	*ALAD*	9q32	Idem, second step
Hydroxymethylbilane synthase	*HMBS*	11q23.3	Idem, third step
Uroporphyrinogen III synthase	*UROS*	10q26.2	Idem, fourth step
Uroporphyrinogen decarboxylase	*UROD*	1p34.1	Idem, fifth step
Coproporphyrinogen oxidase	*CPOX*	3q11.2	Idem, sixth step
Protoporphyrinogen oxidase	*PPOX*	1q23.3	Idem, seventh step
Ferrochelatase	*FECH*	18q21.31	Idem, final step
Heme oxygenase 1	*HMOX1*	22q13.1	Heme catabolism, first step
Tryptophan 2,3‐dioxygenase	*TDO2*	4q32.1	Tryptophan metabolism,heme protein
Indoleamine 2,3‐dioxygenase	*IDO1*	8p12‐p11	Tryptophan metabolism,heme protein
Interleukin 6	*IL6*	7p21‐p15	Inflammation
Tumor necrosis factor	*TNF*	6p21.3	Inflammation, apoptosis
Forkhead box O1	*FOXO‐1*	13q14.1	Energy, insulin signaling
Insulin‐like growth factor‐1	*IGF‐1*	12q23.2	Energy, biomarker of malnutrition
Peroxisome proliferator‐activated receptor gamma coactivator 1‐alpha	*PGC‐1α*	4p15.2	Glucose metabolism, induction of acute porphyria by fasting
House‐keeping genes			
Peptidylprolyl isomerase A (cyclophilin A)	*PPIA*	7p13	
Actin, beta	*ACTB*	7p22.	
TATA box binding protein	*TBP*	6q27	
Beta‐2‐microglobulin	*B2M*	15q21‐q22.2	
Ribosomal protein, large P0	*RPLP0*	12q24.2	
Hypoxanthine guanine phosphoribosyl transferase I	*HPRT1*	Xq26.2	
Transferrin receptor (p90, CD71)	*TFRC*	3q29	
Glucuronidase, beta	*GUSB*	7q11.2	

*Note*: The house‐keeping genes analyzed for normalization are shown at the bottom of the list.

## 2 CASE REPORT

A 36‐years‐old female with AIP harbored a R173W mutation in a heterozygous state in the *HMBS* gene. She presented a history of active disease since the age of 21 years, with recurrent severe neurological attacks over 14 years and was treated with hemin (mainly biweekly heme‐arginate, 3 mg/kg). The patient presented significant comorbidities associated with AIP including renal impairment, neuropathy, hypertension, depression, and anxiety. Overproduction of ALA/PBG was not stabilized by the hemin regime and the patient was nearly continuously admitted to the hospital emergency department. In 2018, the patient underwent orthotopic LT. Twenty‐four hours before surgery, the patient received a last single full hemin infusion as prophylaxis for the procedure, being asymptomatic during the procedure. Urine was collected 4 h before LT for ALA/PBG quantitation. Following LT, the biochemical parameters normalized and the acute attacks disappeared. Peripheral sensory neuropathy stabilized, nevertheless radiofrequency ablation of splanchnic nerves was necessary due to persisting chronic abdominal pain. Three years after orthotopic LT, the patient remains alive with a functioning liver graft. This case was included in the case‐series of Lissing et al.^5^

### 2.1 Human liver samples

A small sample of the explanted AIP liver (~2 g) was obtained directly at the time of surgery, placed in Invitrogen RNA*later* stabilization solution (ThermoFisher Scientific, Waltham, MA, USA) to inhibit RNA degradation and immediately frozen and stored at −80°C until RNA extraction. Another liver specimen was sent to the laboratory for pathological examination and a subsample was used for measurement of hepatic iron concentrations.

Five liver samples from healthy living liver donors, all <50 years of age and stored in a Biobank (Hospital Clinic of Barcelona‐IDIBAPS) were used as controls. Samples were obtained immediately after laparotomy and before vascular clamp. All met the following criteria: (a) no past history of liver disease, alcohol abuse, or metabolic syndrome; (b) absence of maintained arterial hypotension after anesthesia induction; (c) normal serum aminotransferase levels; (d) normal liver histology (no fibrosis or inflammation, and steatosis <10%); (e) no use of pharmacological treatments.

The study was performed in accordance with the principles of the Declaration of Helsinki and approved by the Hospital Clinic Ethics Committee (CEIC, 2018). Written consent was obtained from the AIP patient and the healthy liver donors.

### 2.2 Transcripts investigated by qPCR array

Total RNA was extracted from liver samples of patients and controls in the Hospital Clinic of Barcelona using TRIzol reagent. RNA concentrations were assessed in a Nanodrop 1000 spectrophotometer (NanoDrop Technologies) and integrity was determined in a Bioanalyzer 2100 using the RNA 6000 Nano Assay kit (Agilent Technologies, Santa Clara, CA, USA). The concentration and integrity of the RNA duplicates were satisfactory (RNA integrity number values 7.5–7.9). Thereafter, frozen RNAs were sent to Paris (France) in dry ice and a transcriptomic study was performed by AnyGenes® (Sign‐Array Services, Paris, France). The study included (a): examination of RNA concentrations and purity; (b): reverse transcription to cDNA and quality control, and (c): qPCR array analysis. A unique RNA from the AIP patient was analyzed in triplicate.

The concentrations and purity of the RNAs received were assessed using a Nanodrop‐2000 spectrophotometer (Thermo Fisher Scientific). Before qPCR array analysis, a quality control of all cDNA was performed by qPCR in the Light Cycler 480 instrument (Roche Diagnostics).

All qPCR reactions were performed in the Light Cycler 480 instrument (Roche Diagnostics) with 2× Perfect Master Mix SYBR Green (PMS) (AnyGenes).

Sixteen genes were selected for transcriptome analysis by qPCR (Table 1). These included all nine genes encoding enzymes that consecutively regulate the heme‐synthesis and catabolism pathway: *ALAS1*, *ALAD*; *HMBS*; *UROS*; *UROD*; *CPOX*; *PPOX*; *FECH*, and *HMOX1*. Additional genes investigated were interleukin 6 (IL6) and tumor necrosis factor (TNF; related to hepatic inflammation); tryptophan 2,3‐dioxygenase (TDO2) and indoleamine 2,3‐dioxygenase (IDO1; related to tryptophan metabolism) and PPARG coactivator 1 alfa (PGC‐1α); insulin Like growth factor 1 (IGF‐1) and forkhead box O1 (FOXO‐1; related to insulin‐signaling to ALAS1). The expression of the eight house‐keeping genes was determined for normalization (shown in Table 1). Finally, the normalization step was performed using the crossing point values (Cp) mean of five house‐keeping genes (*PPIA*, *TBP*, *RPLPO*, *HPRT1*, and *GUSB*) all highly stable in all samples (controls and AIP).

### 2.3 Complementary analyses

Iron concentrations in the liver tissue were determined by flameless atomic absorption spectrometry (FLAAS) using the AAnalyst 200 AA spectrometer of Perkin‐Elmer (Waltham, MA, USA) Urinary heme‐precursors were analyzed using the ALA/PBG column test (BioRad GmbH, Munich, Germany). Urinary porphyrins were analyzed by high‐performance liquid chromatography.

### 2.4 Statistical analysis

Statistical analyses were performed using the R computing environment (version 4.1.3). The Shapiro–Wilk test was used to assess the normal distribution of the Cp values obtained by PCR and the Levene test was used to check homoscedasticity. Logarithmic transformations were performed. These data were used for two different calculations: (a) analysis of variance (ANOVA) and Bonferroni post hoc tests (pairwise Student's *t*‐test) were used to statistically evaluate between‐gene expression differences in the controls, and (b) the patient Cp values of each gene were compared with those of the five controls through a *Z*‐score calculation. The *Z*‐score was calculated with the formula *Z*‐score = (raw value of AIP patient – mean of the five controls)/standard deviation of controls.

## 3 RESULTS

PBG and ALA concentrations in a urine sample obtained 4 h pre‐LT were 38 and 15 mmol/mol creatinine, respectively (normal PBG < 1; ALA < 5). Total urinary porphyrins were 173 mmol/mol creatinine (normal <35) with a preponderance (70%) of coproporphyrin‐III isomer. Post‐LT urine analyses showed consistent normalization of heme‐precursors and porphyrins.

Pathological examination of the explanted liver showed no evidence of fibrosis. Perls stain showed the presence of iron deposition within hepatocytes and Kupffer cells, which was more abundant in the periportal and mid‐zonal regions. However, iron tissue concentrations determined by FLAAS were normal (1.18 μg/mg dry weight; normal <1.6), thereby demonstrating that the large amounts of hemin infusions received by this young female did not induce iron‐overload, despite this being a frequent outcome in AIP patients with long‐term hemin regimes.^5^

The concentration and purity of the RNA was assessed in‐house (before transport) and after receipt by AnyGenes. The A260/280 ratios were all between 1.88 and 2.03, thus ruling‐out RNA degradation during transport. The cDNA quality controls performed by qPCR confirmed the excellent condition of the samples. Normal expression of the eight house‐keeping genes confirmed gene integrity.

**FIGURE 1:**
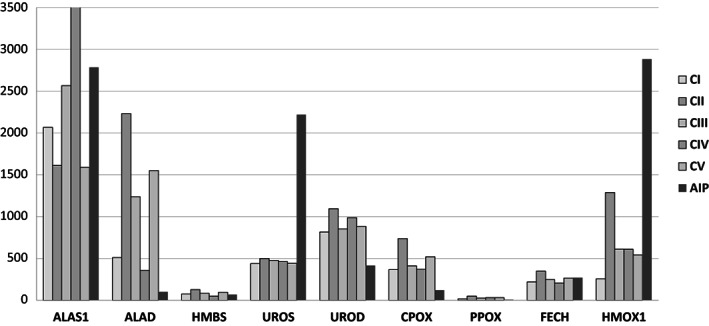
Normalized gene expression in a liver with AIP versus five control healthy livers. The Y axis shows the Cp values (number of RNA copies estimated by qPCR, each value being the mean of triplicate measurements). Each column corresponds to a healthy control (*N* = 5; CI → V, left columns) or the AIP patient (right column). ANOVA and Bonferroni post hoc tests allowed the identification of significant between‐gene expression differences in the five healthy controls. Notably: *ALAS1* versus *HMBS* (*p* < 0.0001); *ALAS1* versus *CPOX* (*p* < 0.0001); *ALAS1* versus *PPOX* (*p* < 0.0001). Only the genes encoding enzymes that catalyze the heme‐synthesis and catabolism pathway ( *ALAS1* → *HMOX1* ) are shown. All the genes studied are shown in Table 2.

The Cp values obtained by PCR assays for all the genes are shown in Table 2. The results illustrate two main types of differences, the first being the between‐gene differences within the healthy controls. Strikingly, the first two genes of the heme‐synthesis pathway showed high mRNA levels compared to the consecutive *HMBS* gene and other genes downstream (*PPOX, FECH*; Figure 1). Differences were more accentuated between *ALAS1 and HMBS* (*p* < 0.0001). Expression of the *TDO2* gene in the controls was very high compared to other genes studied (*TDO2* vs. *ALAS1* > 5 fold, Table 2). The second difference is that the transcription levels of seven genes were significantly different in the AIP‐L compared to the controls. Transcripts of *HMOX1* and *UROS* were significantly increased (*Z*‐scores 5.8 and 67.9, respectively) whereas transcripts of *UROD*; *CPOX*, *PPOX*, and *TDO2* were significantly decreased (Table 2 and Figure 1). Remarkably, the amount of transcripts from *ALAS1*, the gene encoding for the first enzyme of the heme‐synthesis pathway, was within the range of the controls.

**TABLE 2:** qPCR array results

	Healthy controls (*N* = 5)		AIP patient (*N* = 1)	Patient vs. controls	
Gene	Mean	Standard deviation	Measure	Fold‐change	*Z*‐score
*ALAS1*	2444	1156	2784	1.14	0.29
*ALAD*	1178	771	102	−11.57	−1.4
*HMBS*	86	29	68	−1.27	−0.63
*UROS*	464	26	2219	4.78	67.89
*UROD*	928	113	417	−2.23	−4.52
*CPOX*	482	156	120	−4	−2.32
*PPOX*	31	12	6	−5.37	−2.05
*FECH*	257	56	269	1.05	0.22
*HMOX1*	662	379	2882	4.35	5.85
*TDO2*	12 881	2864	5960	−2.16	−2.42
*IDO1*	19	7	19	1.01	0.04
*IL6*	1.6	1.7	1.7	1.01	0.01
*TNF*	12	4	25	2.1	3.33
*FOXO‐1*	422	276	179	−2.36	−0.88
*IGF‐1*	689	230	874	1.27	0.81
*PGC‐1α*	888	389	961	1.08	0.19

*Note*: Left column: gene transcripts analyzed by qPCR (not including house‐keeping genes). Gene expression was normalized using the mean of Cp values of five stable house‐keeping genes and defined by 2^(−dCp) × 1000 values. Second and third columns: Normalized gene expression of the five healthy controls, showing mean and standard deviation of the values obtained in each patient. Fourth column: Cp values obtained from a unique liver sample from an AIP patient. Fifth column: fold‐change of the patient (single Cp value) versus the controls (mean of five Cp values). Right column: *Z*‐score of the patient Cp values.

## 4 DISCUSSION

The number of mRNA transcripts in liver tissue measured by qPCR mainly reflects the amount of gene expression. However, other factors (i.e., speed of post‐transcriptional mRNA decay) can also ultimately determine the qPCR results. Nonetheless, in the discussion below we assume that gene expression is the main determinant of the mRNA measured.

The results in healthy controls showed important differences in between‐gene expression. Remarkably, *ALAS1* showed a high level of expression in all five donors compared to other more distal genes in the heme biosynthetic pathway (*HMBS*, *PPOX*, and *FECH*). Compared with the other enzymes of the pathway, *ALAS1* shows the lowest relative capacity in the liver and is rate‐limiting.^8^ Phenobarbital and other drugs rapidly induce gene transcription to increase the synthesis of P450s to cope with metabolic demands.^9^ Thus, unless anesthesia during surgery induced *ALAS1*, the results indicate a high constitutive transcription rate, possibly to compensate for the lower *V*_max_ of the enzyme. Similarly, *ALAD* also showed a robust constitutive transcription. In striking contrast, the *HMBS* gene showed low expression in all five healthy controls. The HMBS enzyme has also weak catalytic capacity,^9^ which is critical since decreased activity in mutation‐carriers combined with *ALAS1* up‐regulation induce acute porphyria attacks.^2^ Heme‐precursors accumulation occur in other forms of acute porphyria (i.e., variegate porphyria) and rare intoxications (i.e., lead poisoning) but is seldom found in normal individuals, even if *ALAS1* expression is strongly induced by internal or external stimuli.^2^, ^9^ Thus, it is remarkable that while being consecutive to highly expressed *ALAS1*/*ALAD* genes, a low‐expressed *HMBS* gene encodes an enzyme that effectively prevents PBG/ALA accumulation in a healthy liver. Thus, our results suggest fine tuning of the regulation of the whole chain in normal individuals with transcriptomics playing an important role.

mRNAs determined in the AIP‐L showed several significant differences compared to the healthy controls. Some of these results may be interpreted as follows.

The lack of increase of *ALAS1*‐mRNA in the AIP‐L is of note, considering that *ALAS1* up‐regulation is a hallmark of acute porphyria attacks. However, the patient did not show acute symptoms the days before LT and prophylactic hemin administered immediately prior to LT apparently reduced *ALAS1*‐mRNA by the well‐known negative feed‐back mechanisms.^2^ Yasuda et al.^6^ also reported normal *ALAS1*‐mRNA levels in the explant. This suggests that post‐hemin transcriptomics differ from what would presumably be found during an acute attack and when an intervention (aimed at reducing ALAS1 activity is effective.

*ALAD*‐mRNA was low in the AIP‐L, thus suggesting gene down‐regulation. This could theoretically induce ALA accumulation. Therefore, even if acute AIP attacks originate from the coinciding of *ALAS1* up‐regulation and *HMBS* deficiency,^2^ an additional role of *ALAD* (i.e., gene down‐regulation induced by metabolic stress) contributing to ALA accumulation should be further investigated.

Remarkably, no up‐regulation of *HMBS* was observed in the AIP‐L despite the patient presenting a germinal pathogenic variant that reduces HMBS enzymatic activity to ≈50%.

*UROS* was significantly up‐regulated in the AIP‐L. Interestingly, AIP patients usually show increased urinary concentrations of uroporphyrin type I and III isomers, originating from spontaneous PBG condensation. However, increased type‐III isomers could also originate, in part, from in vivo enzymatic formation.^1^ Thus, increased *UROS* gene expression could facilitate the physiological formation of uroporphyrin III.

Differently, *CPOX*‐mRNA showed low values in the AIP‐L suggesting gene down‐regulation. Interestingly, pre‐LT urine analysis of the patient showed increased levels of the coproporphyrin III isomer, a common feature found in the urine of AIP patients.^1^

Finally, increased *HMOX1*‐mRNA in our AIP‐L was expected, since both the American and French studies^6^, ^7^ reported increased transcription. This gene encodes for heme oxygenase‐1 (HO‐1) a major enzyme for the catabolism of heme. *HMOX1* transcription may be enhanced by cellular free heme through Bach/NrF2 regulatory elements.^10^ Thus, pre‐LT hemin infusions would most likely explain the increased *HMOX1‐mRNA*. Our results reinforce the concept that heme auto‐enhanced catabolism through *HMOX1* may explain the short‐lived clinical effect of hemin, eventually leading to *ALAS1* re‐induction.

We found only slightly increased *TNF*‐mRNA and normal *IL‐*6‐mRNA values; thus, even though inflammation has been described as being associated with recurrent AIP,^7^ we found no clear transcriptomic evidence of this association in this patient.

*TDO2* and *IDO1* encode heme‐dependent enzymes and both catalyze the metabolism of tryptophan to kynunerine. We found a high level of *TDO2* expression in the controls confirming that this gene is strongly expressed in the liver (Table 2). A previous study^11^ reported that AIP patients present an increased kynurenine/tryptophan ratio in urine, suggesting activation of the hepatic kynurenine pathway. In this case, our results suggest down‐regulation of the *TDO2* gene in the liver. A reasonable explanation would be that hemin administered to the patient before LT and replenishment of the heme pool modified *TDO2* gene expression. However, this requires confirmation in animal models

As for the genes involved in insulin signaling (*PGC‐1α*, *IGF‐1*, and *FOXO‐1*), we only found a slight decrease in *FOXO1*‐mRNA levels. This suggests that even if these genes are associated with fasting, malnutrition, and acute porphyria attacks, the AIP patient presented an acceptable nutritional status at the time of LT.

In summary, our study of the liver of a recurrent AIP patient showed several transcriptomic changes related to heme synthesis (*UROS*), degradation (*HMOX1*), and utilization (*TDO2*). Possibly, pre‐LT hemin administration normalized *ALAS1* transcription. In addition, the study of the healthy controls showed large differences between the transcription of the various genes with, for example, *ALAS1* being remarkably highly expressed compared to other genes downstream from the heme‐synthesis pathway (*HMBS*, *CPOX*, and *PPOX*). This offers a glimpse of how the expression of these genes may be finely tuned to ensure sufficient heme formation in a normal liver without inducing accumulation of heme‐precursors.

## CONFLICT OF INTEREST

Jordi To‐Figueras has received honorarium for participation on advisory boards of Alnylam Pharmaceuticals (Spain) and Recordati Rare Diseases. Esther Titos, Paula Aguilera, Alba Díaz, Javier Muñoz‐Luque, Irene Madrigal, Celia Badenas, Mercè Torra, Constantino Fondevila, and Jordi Colmenero declare no conflict of interest.

## ETHICS STATEMENT

All procedures were performed in accordance with the ethical standards of the responsible committee on human experimentation (institutional and national) and with the Declaration of Helsinki of 1975 (revised in 2000). The ethical committee of our institution approved the project (Hospital Clinic of Barcelona, CEIC. HCB 2017/0902) after the AIP patient provided signed written consent and healthy living liver donors accepted transcriptomic analyses of liver samples stored in the institutional Biobank (Institut de Recerca Biomèdica August Pi Sunyer). Confidentiality of the results was guaranteed by hospital protocols and databases.

## Data Availability

The manuscript has no associated data.
